# Dosimetric comparison between helical tomotherapy and volumetric modulated arc-therapy for non-anaplastic thyroid cancer treatment

**DOI:** 10.1186/s13014-014-0247-x

**Published:** 2014-11-26

**Authors:** Jonathan Khalifa, Laure Vieillevigne, Sabrina Boyrie, Monia Ouali, Thomas Filleron, Michel Rives, Anne Laprie

**Affiliations:** Department of Radiation Oncology, Institut Claudius Regaud, Institut Universitaire du Cancer de Toulouse, Oncopôle – 1, avenue Irène Joliot Curie, 31059 Toulouse, France; Department of Medical Physics, Institut Claudius Regaud, Institut Universitaire du Cancer de Toulouse, Oncopôle – 1, avenue Irène Joliot Curie, 31059 Toulouse, France; Department of Biostatistics, Institut Claudius Regaud, Institut Universitaire du Cancer de Toulouse, Oncopôle – 1, avenue Irène Joliot Curie, 31059 Toulouse, France; Université Toulouse III Paul Sabatier, Toulouse, F-31000 France; INSERM, UMR825, Toulouse, 24 F-31059 France

**Keywords:** Volumetric modulated arctherapy, Helical tomotherapy, Non-anaplastic thyroid cancer, Dosimetric comparison

## Abstract

**Background:**

To evaluate and compare dosimetric parameters of volumetric modulated arctherapy (VMAT) and helical tomotherapy (HT) for non-anaplastic thyroid cancer adjuvant radiotherapy.

**Methods:**

Twelve patients with non-anaplastic thyroid cancer at high risk of local relapse received adjuvant external beam radiotherapy with curative intent in our institution, using a two-dose level prescription with a simultaneous integrated boost approach. Each patient was re-planned by the same physicist twice using both VMAT and HT. Several dosimetric quality indexes were used: target coverage index (proportion of the target volume covered by the reference isodose), healthy tissue conformity index (proportion of the reference isodose volume including the target volume), conformation number (combining both previous indexes), Dice Similarity Coefficient (DSC), and homogeneity index ((D2%-D98%)/prescribed dose). Dose-volume histogram statistics were also compared.

**Results:**

HT provided statistically better target coverage index and homogeneity index for low risk PTV in comparison with VMAT (respectively 0.99 vs. 0.97 (p = 0.008) and 0.22 vs. 0.25 (p = 0.016)). However, HT provided poorer results for healthy tissue conformity index, conformation number and DSC with low risk and high risk PTV. As regards organs at risk sparing, by comparison with VMAT, HT statistically decreased the D2% to medullary canal (25.3 Gy vs. 32.6 Gy (p = 0.003)). Besides, HT allowed a slight sparing dose for the controlateral parotid (Dmean: 4.3 Gy vs. 6.6 Gy (p = 0.032)) and for the controlateral sub-maxillary gland (Dmean: 29.1 Gy vs. 33.1 Gy (p = 0.041)).

**Conclusions:**

Both VMAT and HT techniques for adjuvant treatment of non-anaplastic thyroid cancer provide globally attractive treatment plans with slight dosimetric differences. However, helical tomotherapy clearly provides a benefit in term of medullary canal sparing.

## Background

Thyroid cancer is the 18th most common cancer in Europe, with around 53000 new cases diagnosed in 2012 (2% of the total) and with reported increases in incidence between 1973 and 2002 from 5.3% (Switzerland) to 155.6% (among French males) [1].

Differentiated thyroid cancers (DTC) comprise the vast majority of all thyroid cancers (90%, including 80% of papillary and 10% of follicular thyroid cancers), followed by medullary thyroid cancers (MTC) (4%), poorly differentiated thyroid cancers (PDTC) (4%), and anaplastic thyroid cancers (ATC) (1-2%) [2,3].

Tumor histology is a critical determinant of patient outcomes: DTC is associated with the best survival rates (10-year relative survival: 93% for papillary carcinoma and 85% for follicular carcinoma), and ATC with the poorer outcomes (10-year relative survival: 14%). PDTC and MTC have intermediate prognosis [4]. Besides, several histological subtypes of papillary carcinoma have been described with poorer outcomes compared to classical papillary carcinoma (diffuse sclerosing and tall cell variants) [5]. Lymph node metastases represent another strong prognosis factor of recurrence and reduced survival especially in patients aged >45 years [6–9]. Contrary to follicular carcinoma, lymph nodes involvement is commonly described in classic papillary carcinomas (56%) and more frequently in aggressive variants (respectively 72% and 67% with diffuse sclerosing and tall cell variants) and in MTC (55-75%) [5,10,11]. Several other poor prognosis factors have been established with DTC including age >40-60 years, presence and extent of extrathyroidal extension (T3, T4), tumor size >1 cm, extent of post-surgical residual disease, or absence of radioactive iodine uptake [6,12–14].

In non metastatic stages, the main and first step of treatment consists of surgery. In case of DTC >1 cm and/or presence of one of the previous high risk features, surgery is followed by radioactive iodine (RAI) therapy [15].

The role of external beam radiotherapy (EBRT) in thyroid cancer has been studied in many retrospective studies, and only a subset of patients at high risk of local recurrence may benefit from adjuvant EBRT [15–18].

Due to cervico-mediastinal localization of these cancers, with several complicated-shape organs at risk around the thyroid, EBRT can be difficult to plan. Major improvements in radiation therapy in the last decade have first led to fixed-field intensity-modulated radiotherapy (IMRT), allowing dose escalation on the target volume as well as protection of organs at risk (OAR). In the field of thyroid cancer, only few clinical or dosimetric data exist about IMRT [19–22]. These data suggest that compared to 3D conformational radiotherapy, IMRT improves PTV coverage and spinal cord sparing, and is associated with less frequent late morbidity but with no impact on survival outcomes. Further improvements in treatment optimization and planning treatment led to better dosimetric performance with Volumetric Modulated Arctherapy [23] (RapidArc for Varian Medical System® and VMAT for Elekta®), or Helical Tomotherapy [24] (Accuray®).

The prolonged survivals obtained for locally advanced thyroid cancer after adjuvant EBRT [13,16,20,25,26] should lead us to choose the technique allowing the best OAR sparing to avoid late toxicities, with optimal planning target volume coverage. Nevertheless, no comparison between these two last techniques exists in this way in the field of thyroid cancer adjuvant radiotherapy.

Therefore, the aim of this retrospective study was thus to compare volumetric modulated arc-therapy (VMAT RapidArc from Varian Medical System®) and Helical Tomotherapy (HT) for adjuvant treatment of non-anaplastic thyroid cancer, in terms of dose distribution to the Planning Target Volume and OAR protection.

## Methods

### Population

From March 2011 to December 2013, 12 patients with histologically proven non-anaplastic thyroid cancer were consecutively irradiated by VMAT or HT at our institution in a curative intent.

Patient’s characteristics are summarized in Table 1.

**Table 1:** **Patient and tumor characteristics**

**Patient**	**Pathological subtypes**	**Subsites**	**TNM**	**Neck levels involved**	**Nodal extracapsular spread**	**Surgical margin status**	**PTV 63**	**PTV 54**
							**Volume (cm** ^**3**^ **)/**	**Volume (cm** ^**3**^ **)/**
							**Height (cm)**	**Height (cm)**
1	Medullary	Right lobe	T3N1a	R: VI	Y	R1	298.55/13.26	534.84/18.42
2	Medullary	Left lobe	T4aN1b	R: VI	Y	R1	490.76/14.54	954.89/20.62
				L: II, III, V, VI				
3	Vesicular	Bilateral	T4aN0	-	-	R2	254.54/11.36	500.39/13.36
4	Medullary	Right lobe	T2N1b	R: IIA, III, IV, V, VI	Y	R0	-	641.07/20.78
				L: VI				
5	Medullary	Bilateral	T3N1b	L: IIA, VI	Y	R1	93.3/10.62	262.36/18.33
				R: VI				
6	Medullary	Right lobe	T2N1b	R: III, IV, VI	Y	R0	206.97/13.37	545.01/18.66
7	Papillary	Bilateral	T4aN1a	R: VI	Y	R1	124.11/5.9	-
8	Papillary	Left lobe	T4aN1b	L: III, IV, VI	Y	R1	39.39/4.24	247.87/14.92
9	Medullary	Right lobe	T3N1b	R: III, VI	Y	R1	83.52/8.59	300.35/16.07
10	Medullary	Left lobe	T3N1b	L: IV, VI	Y	R1	405.82/22.43	596.87/22.43
				R: VI, VII				
11	Papillary	Bilateral	T3N1b	R: IV, VI	Y	R0	51.36/6.02	361.89/17.35
12	Papillary	Left lobe	T4aN1a	L: VI	N	R1	95.08/8.44	201.39/13.12
Average							194.85/10.8	467.91/17.64

R = right; L = left; R0 = no residual tumor; R1 = microscopic residual tumor; R2 = macroscopic residual tumor; Y = yes; N = No.

### Therapeutic sequence and indications of EBRT

All the patients underwent external beam radiotherapy (EBRT) in first-line treatment after initial surgery and radioactive iodine therapy for all non medullary carcinomas (100 mCi). The initial surgery consisted of total thyroidectomy with lymph node dissection for all 12 patients (central compartment neck dissection for all patients and lateral neck dissection for ten patients).

No patient received chemotherapy.

Indication of EBRT was as follows: node extracapsular spread and/or microscopic or macroscopic residual disease following surgery.

### Planning target volumes, organs at risk delineation and dose prescription

High risk of recurrence clinical target volume (CTV) included primary tumor bed with microscopic positive margins, residual gross tumor at first post-surgical assessment, or areas of nodal extra-capsular spread, and was treated with high dose EBRT. Low risk CTV included primary and nodal micro-metastatic disease (including level VII nodes, according to the classical patterns of relapse after radiotherapy [25]), and was treated with low dose EBRT.

Level II nodes were included in low risk CTV in nine patients and in high risk CTV in two patients. Levels III-IV-VI nodes were included in high risk CTV in seven patients.

PTV was built by 3D-automatic expansion of CTV with a 3 millimeters margin.

Among the twelve patients considered: ten patients received a high dose/low dose prescription, one patient received only a high dose prescription and one patient received only a low dose prescription. High risk PTV was treated according to simultaneous integrated boost technique to deliver 63 Gy by 30 fractions of 2.1 Gy, whereas low risk PTV was treated with 54 Gy by 30 fractions of 1.8 Gy.

According to ICRU 83 report, the dose prescription referred to the median dose (D50%).

The OAR included: medullary canal, mandible, parotid glands, sub-maxillary glands, larynx, oral cavity, esophagus and brachial plexus.

Finally, healthy tissue (non-tumor tissue volume) was defined as:

- Volume between the vertex and the apex of xyphoïd process – (PTV63 U PTV54)

All contours were approved by a single physician widely experimented in head and neck cancers radiation oncology.

### Dose volume constraints and inverse treatment planning

Treatment planning was recalculated for each patient with both VMAT and HT by the same physicist (experienced in both techniques).

All the VMAT treatment plans were computed using the Eclipse TPS v8.9 (Varian Medical Systems, Inc., Palo Alto, CA). Dose calculation was performed using the Anisotropic Analytical Algorithm (AAA) with a calculation grid resolution of 2.5 mm. Treatment plans were optimized for a Varian 2100 iX clinac equipped with a Millenium 120 MLC. Each VMAT plan was designed using two 6 MV photon coplanar arcs of 360°: clockwise (CW) and counterclockwise (CCW) divided in 177 control points. The collimator angle was equal to 15° and 345° for the CW and the CCW arcs respectively. The optimization was based on the PRO II algorithm. Normal Tissue Objective (NTO) algorithm was used in the VMAT planning in order to minimize the dose deposited outside of the PTV. The NTO was used with a distance from the target of 3 mm, a start dose of 100%, an end dose of 30% and a fall off of 0.1. The maximal dose rate was set to 600 MU/min.

HT used a helical slice 6 MV photon beam, modulated in intensity using binary MLCs. For all plans, we used a field width of 2.5 cm with a pitch value of 0.287 and a nominal modulation factor of 2.5. Plans were optimized using the Tomotherapy Hi-Art TPS, version 4.2.1 (Tomotherapy Inc. Madison, WI). The dose distribution for each beamlet was calculated using a convolution/superposition algorithm. The optimization process used the least mean squares method to minimize the objective function.

During planning, the first objective was to ensure PTV coverage. The criteria for plan validation were: more than 95% of the prescribed dose to more than 95% of the PTV, more than 90% of the prescribed dose to more than 98% of the PTV, and less than 107% of the prescribed dose to less than 2% of the PTV. The D50% had to be as close as possible to 54Gy and 63Gy for PTV54 Gy and PTV63 Gy respectively.

The secondary objective was to minimize OAR doses as much as possible. For esophagus and brachial plexus, no optimization criteria were defined, but sparing was allowed by the NTO algorithm in VMAT and by non-anatomical optimization “dummy volumes” in HT.

After each planning, we made sure that it was reasonably deliverable.

The optimization objectives for PTV and OAR with respective priorities and the criteria for plan validation are summarized in Tables 2 and 3.

**Table 2:** **Dose-volume constraints for PTVs and OARs used for optimization**

	**VMAT**		**Helical tomotherapy**	
**Structures**	**Optimization objectives**	**Optimization priorities**	**Optimization objectives**	**Optimization priorities**
PTV _63 Gy_	D_50%_ = 63 Gy	Very High	D_min_ = 63 Gy	Very high
	D_min_ > 100% D_presc HR_	Very High	D_50%_ = 63 Gy	Very high
	D_max_ < 102% D_presc HR_	Very High	D_max_ = 63 Gy	Very high
PTV _54 Gy_	-	-	D_min_ = 54 Gy	High
			D_50%_ = 54 Gy	High
			D_max_ = 54 Gy	High
PTV _54 Gy_ – PTV _63 Gy_	D_min_ > 100%D_presc LR_	Very High	-	-
	D_max_ < 95% D_presc HR_	High		
	D_5%_ < 105%D_presc LR_	High		
PTV _54 Gy_ –(PTV _63 Gy_ +1 cm)	D_max_ < 95%D_presc LR_	High	-	-
PRV medullary canal	D_max_ < 35Gy	High	D_max_ < 30 Gy	High
	D_2%_ < 33 Gy		D_2%_ < 28Gy	
Ipsilateral parotid gland - PTVs	V_10Gy_ < 45%	Medium	V_15Gy_ < 45%	Medium
	V_25Gy_ < 30%		V_30Gy_ < 30%	
	V_40Gy_ < 15%		V_45Gy_ < 15%	
			D_max_ < 50Gy	
Controlateral parotid gland - PTVs	V_10Gy_ < 45%	High	V_15Gy_ < 45%	High
	V_25Gy_ < 30%		V_30Gy_ < 30%	
	V_40Gy_ < 15%		V_45Gy_ < 15%	
			D_max_ < 50Gy	
Mandible - PTVs	D_5%_ < 55Gy	Medium	D_max_ < 65Gy	Medium
	D_50%_ < 30Gy		D_5%_ < 55Gy	
			D_50%_ < 30Gy	
Larynx - PTVs	D_max_ < 55Gy	Medium	D_max_ < 55Gy	Medium
	D_50%_ < 30Gy		D_50%_ < 30Gy	
	D_95%_ < 15Gy		D_95%_ < 15Gy	
Oral cavity - PTVs	D_max_ < 55Gy	Medium	D_max_ < 55Gy	Medium
	D_50%_ < 30Gy		D_50%_ < 30Gy	
	D_95%_ < 15Gy		D_95%_ < 15Gy	
Ipsilateral sub-maxillary gland - PTVs	D_30%_ < 50Gy	Medium	D_max_ < 55Gy	Medium
	D_50%_ < 30Gy		D_30%_ < 50Gy	
			D_50%_ < 30Gy	
Controlateral sub-maxillary gland - PTVs	D_30%_ < 50Gy	High	D_max_ < 55Gy	High
	D_50%_ < 30Gy		D_30%_ < 50Gy	
			D_50%_ < 30Gy	

D_x%_ = dose received by x% of structure volume; V_xGy_ = percent structure volume of xGy.

D_presc_ _HR_ = prescribed dose on high risk PTV = 63 Gy; D_presc LR_ = prescribed dose on low risk PTV =54 Gy.

**Table 3:** **Criteria for plan validation**

**Structures**	**Dose-constraint**
PTV _63 Gy_	D_50%_ = 63 Gy
	D_95%_ > 95%D_presc HR_
	D_98%_ > 90%D_presc HR_
	D_2%_ **<** 107%D_presc HR_
PTV _54 Gy_	D_95%_ > 95%D_presc LR_
	D_98%_ > 90%D_presc LR_
PRV medullary canal	D_max_ < 45Gy
Parotid glands*	Dmean < 26Gy
	V_15Gy_ < 65%
	V_30Gy_ < 45%
	V_45Gy_ < 24%
Mandible	D_mean_ < 30Gy
	D_max_ < 70Gy
Larynx	D_mean_ < 30Gy
	D_max_ < 55Gy
Oral cavity	D_mean_ < 30Gy
	D_max_ < 55Gy
Sub-maxillary glands*	D_mean_ < 40Gy

*At least one.

### Dosimetric parameters

Plans quality was first assessed according to simple criteria from ICRU 83: near-minimal dose (D98%), near-maximal dose (D2%) and median dose (D50%).

Other indexes were used to compare both treatment techniques:

1. Dose conformity [27]:
  Target coverage index (TCov-I) corresponds to the proportion of target volume covered by the reference isodose (95% of the prescribed dose), regardless of healthy tissue [28]:TCov ‐ I = VT_IR_/VT
    with VT: target volume (ie PTV),
    and VT_IR_: target volume covered by the reference isodose volume (95% of the prescribed dose).
  Healthy tissue conformity index (HTConf-I) corresponds to the proportion of the reference isodose volume including the target volume. Indirectly, it refers to the volume of healthy tissue included in the reference isodose [28]:
    $$ \mathrm{HTConf}\hbox{-} \mathrm{I} = \mathrm{V}{\mathrm{T}}_{\mathrm{IR}}/{\mathrm{V}}_{\mathrm{IR}} $$
    with V_IR_: reference isodose volume (95% of prescribed dose)
  Conformation number (CN) is a global index which provides information as well on tumor coverage as on protection of healthy tissue [29]:
    $$ \mathrm{C}\mathrm{N} = \left(\mathrm{V}{\mathrm{T}}_{\mathrm{IR}}/\mathrm{V}\mathrm{T}\right)\ \mathrm{x}\ \left(\mathrm{V}{\mathrm{T}}_{\mathrm{IR}}/{\mathrm{V}}_{\mathrm{IR}}\right) $$
  Dice Similarity Coefficient (DSC) is defined in ICRU 83 report as the ratio between twice the tumor volume covered by the reference isodose and the sum of tumor volume and reference isodose volume:
    $$ \mathrm{D}\mathrm{S}\mathrm{C} = 2\mathrm{x}\mathrm{V}{\mathrm{T}}_{\mathrm{IR}}/\ \left(\mathrm{V}\mathrm{T} + {\mathrm{V}}_{\mathrm{IR}}\right) $$
  The ideal value of these four indexes is 1.
2. Dose homogeneity:
  $$ \mathrm{Homogeneity}\ \mathrm{index}\ {(HI)}_{ICRU} = \left(D2\%-D98\%\right)/D50\% $$
  The ideal value of HI is 0.
3. For healthy tissue, we defined integral dose (ID) to the non tumor-tissue volume (NTID) as follows [30,31]:
  $$ \mathrm{NTID} = \mathrm{ID}\ \mathrm{bod}{\mathrm{y}}_{\Big(\mathrm{between}\ \mathrm{the}\ \mathrm{vertex}\ \mathrm{and}\ \mathrm{the}\ \mathrm{apex}\ \mathrm{of}\ \mathrm{xyphoid}\ \mathrm{process}}\Big)\ \hbox{--}\ \mathrm{ID}\ \mathrm{P}\mathrm{T}\mathrm{V}{\mathrm{s}}_{\left(\mathrm{P}\mathrm{T}\mathrm{V}54\ \mathrm{U}\ \mathrm{P}\mathrm{T}\mathrm{V}63\right)} $$
  where:
  $$ \begin{array}{c}\mathrm{ID}{{}_{\mathrm{structure}}}_{\mathrm{S}}\;\left(\mathrm{Joules}\right) = {{\mathrm{D}}_{\mathrm{m}\mathrm{ean}}}_{\mathrm{S}}\;\left(\mathrm{Gy}\right)\ \mathrm{x}\ \mathrm{Volum}{\mathrm{e}}_{\mathrm{S}}\;\left(\mathrm{c}{\mathrm{m}}^3\right)\ \mathrm{x}\ \mathrm{densit}{\mathrm{y}}_{\mathrm{S}}\;\left(\mathrm{kg}.\mathrm{c}{\mathrm{m}}^{\hbox{-} 3}\right),\ \\ {} = {{\mathrm{D}}_{\mathrm{m}\mathrm{ean}}}_{\mathrm{S}}\;\left(\mathrm{Gy}\right)\ \mathrm{x}\ \mathrm{Volum}{\mathrm{e}}_{\mathrm{S}}\;\left(\mathrm{c}{\mathrm{m}}^3\right)\;\mathrm{with}\ \mathrm{densit}\mathrm{y}\kern0.37em  \approx 1,\ \end{array} $$

### Statistical analysis

Patients’ characteristics were described using median and range for quantitative data, and frequency and percent for qualitative data.

The comparison between the two techniques in the paired population was made using the Wilcoxon signrank test. All tests were two-tailed and p < 0.05 was considered to indicate statistical significance. All statistical analyses were done with STATA 12.0 software (StataCorp. 2011. Stata Statistical Software: Release 12. College Station, TX: StataCorp LP).

This study was approved by the scientific board of the multidisciplinary head-and-neck tumor institutional group.

## Results

The results are summarized in Tables 4 and 5.

**Table 4:** **Median (range) dosimetric results for planning target volumes**

**PTV**	**Dosimetric parameters**	**VMAT**	**Helical Tomotherapy**	**P**
PTV 63	D_2%_ (Gy)	65.4 (64.2-67.0)	64.9 (64.1-66.0)	0.131
	D_50%_ (Gy)	63 (63.0-63.9)	62.9 (62.8-63.1)	0.062
	D_98%_ (Gy)	58.4 (57.4-60.7)	59.3 (57.0-60.7)	0.248
	TCov-I	0.94 (0.90-0.99)	0.96 (0.91-0.99)	0.091
	HTConf-I	0.85 (0.77-0.92)	0.77 (0.66-0.94)	0.016*
	CN	0.78 (0.76-0.87)	0.74 (0.62-0.85)	0.033*
	DSC	0.88 (0.87-0.93)	0.85 (0.77-0.92)	0.033*
	HI	0.11 (0.06-0.15)	0.09 (0.05-0.13)	0.109
PTV 54	D_2%_ (Gy)	64.9 (56.3-66.4)	64.3 (55.0-65.7)	0.109
	D_50%_ (Gy)	57.5 (54.0-62.3)	57.1 (54.0-61.9)	0.168
	D_98%_ (Gy)	51.0 (48.6-52.0)	51.8 (50.5-53.6)	0.016*
	TCov-I	0.97 (0.90-0.99)	0.99 (0.97-1.00)	0.008*
	HTConf-I	0.84 (0.71-0.89)	0.63 (0.51-0.69)	0.003*
	CN	0.76 (0.69-0.82)	0.62 (0.50-0.67)	0.003*
	DSC	0.86 (0.78-0.90)	0.77 (0.67-0.81)	0.003*
	HI	0.25 (0.12-0.29)	0.22 (0.06-0.26)	0.016*

TCov-I = Target Coverage Index; HTConf-I = Healthy Tissue Conformity Index; CN = Conformation Number; DSC = Dice Similarity Coefficient; HI = Homogeneity Index; * = p < 0.05.

**Table 5:** **Median (range) dosimetric results for organs at risk and healthy tissue**

**Organ**	**Dose-volume index**	**VMAT**	**Helical tomotherapy**	**P**
Medullar canal	D_2%_ (Gy)	32.6 (30.1-39.0)	25.3 (23.0-34.6)	0.003*
PRV medullar canal	D_2%_ (Gy)	34.3 (31.6-40.4)	27.2 (24.1-35.1)	0.003*
I parotid gland	V_26Gy_ (%)	31.1 (0–42.6)	33.8 (0–45.9)	0.812
	D_mean_ (Gy)	19.9 (0–24.4)	19.5 (0–25.8)	0.812
C parotid gland	V_26Gy_ (%)	0 (0–49.3)	0 (0–47.3)	0.85
	D_mean_ (Gy)	6.6 (0–28.1)	4.3 (0–27.2)	0.032*
I submaxillary gland	D_mean_ (Gy)	36.1 (15.4-44.6)	34.5 (24.7-44.6)	0.623
C submaxillary gland	D_mean_ (Gy)	33.1 (3.4-41.9)	29.1 (1.5-39.6)	0.041*
Mandible	D_2%_ (Gy)	35.6 (1.4-52.2)	42.6 (0.9-58.0)	0.01*
Oral cavity	D_mean_ (Gy)	17.2 (0.5-27.4)	17.8 (0.5-32.2)	0.075
Larynx	D_2%_ (Gy)	60.8 (49.2-64.9)	60.7 (43.6-65.0)	0.209
	D_mean_ (Gy)	45.9 (10.8-62.5)	39.2 (25.0-62.6)	0.638
Esophagus	D_2%_ (Gy)	60.9 (47.7-63.7)	57.5 (41.6-64.7)	0.049*
I brachial plexus	D_2%_ (Gy)	62.7 (44.1-67.4)	62.5 (46.4-64.6)	0.209
C brachial plexus	D_2%_ (Gy)	54.4 (29.7-58.5)	54.2 (26.8-55.1)	0.308
ID body	(Joules)	138.5 (54.9-242.3)	149.5 (72.5-271.0)	0.008*
NTID	(Joules)	117.0 (47.0-201.6)	124.6 (64.7-226.3)	0.008*

D_x%_ = dose received by x% of structure volume; V_xGy_ = percent structure volume of xGy; I = ipsilateral; C = controlateral.

ID = Integral Dose; NTID = Normal Tissue Integral Dose; * = p < 0.05.

### High risk PTV

In comparison with VMAT, HT provided poorer results for HTConf-I (0.77 vs. 0.85 (p = 0.016)), CN (0.74 vs. 0.78 (p = 0.033)) and DSC (0.85 vs. 0.88 (p = 0.033)).

HT had better TCov-I and HI value than VMAT, however it was not statistically significant (respectively: 0.96 vs. 0.94 (p = 0.091) and 0.09 vs. 0.11 (p = 0.109)).

### Low risk PTV

In comparison with VMAT, HT allowed an improvement in TCov-I and HI: respectively 0.99 vs. 0.97 (p = 0.008) and 0.22 vs. 0.25 (p = 0.016).

On the other hand, we found poorer results with HT for HTConf-I, CN and DSC: respectively 0.63 vs. 0.84 (p = 0.003), 0.62 vs. 0.76 (p = 0.003) and 0.77 vs. 0.86 (p = 0.003).

### Organs at risk and healthy tissue

By comparison with VMAT, HT significantly improved the D2% to medullary canal and to PRV (planning organ at risk volume) medullary canal: respectively 25.3 Gy vs. 32.6 Gy (p = 0.003) and 27.2 Gy vs. 34.3 Gy (p = 0.003).

As for parotid sparing, we failed to find a strong dosimetric benefit with HT compared to VMAT (except a decrease in Dmean to the controlateral parotid with HT: 4.3Gy vs. 6.6Gy (p = 0.032)).

Besides, Dmean to the controlateral submaxillary gland was also decreased with HT (29.1 Gy vs. 33.1 Gy (p = 0.041)).

On the other hand, HT provided a higher D2% to mandible (42.6 Gy vs. 35.6 Gy (p = 0.01)).

Finally, there was higher NTID with HT compared to VMAT (124.6 Joules vs. 117.0 Joules (p = 0.008)).

**Figure 1:**
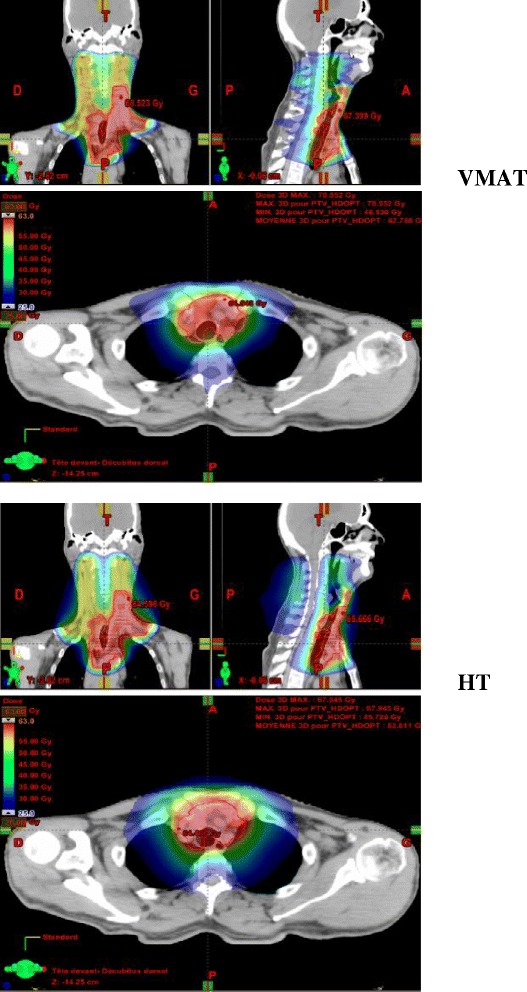
**Dose distribution in color-wash for one representative patient with VMAT (top) and HT (bottom).** Red line: PTV63. This figure shows a color-wash of the dose distribution from 25Gy to 63Gy in VMAT and HT for one patient: a better medullary canal sparing is obtained with HT compared to VMAT.

**Figure 2:**
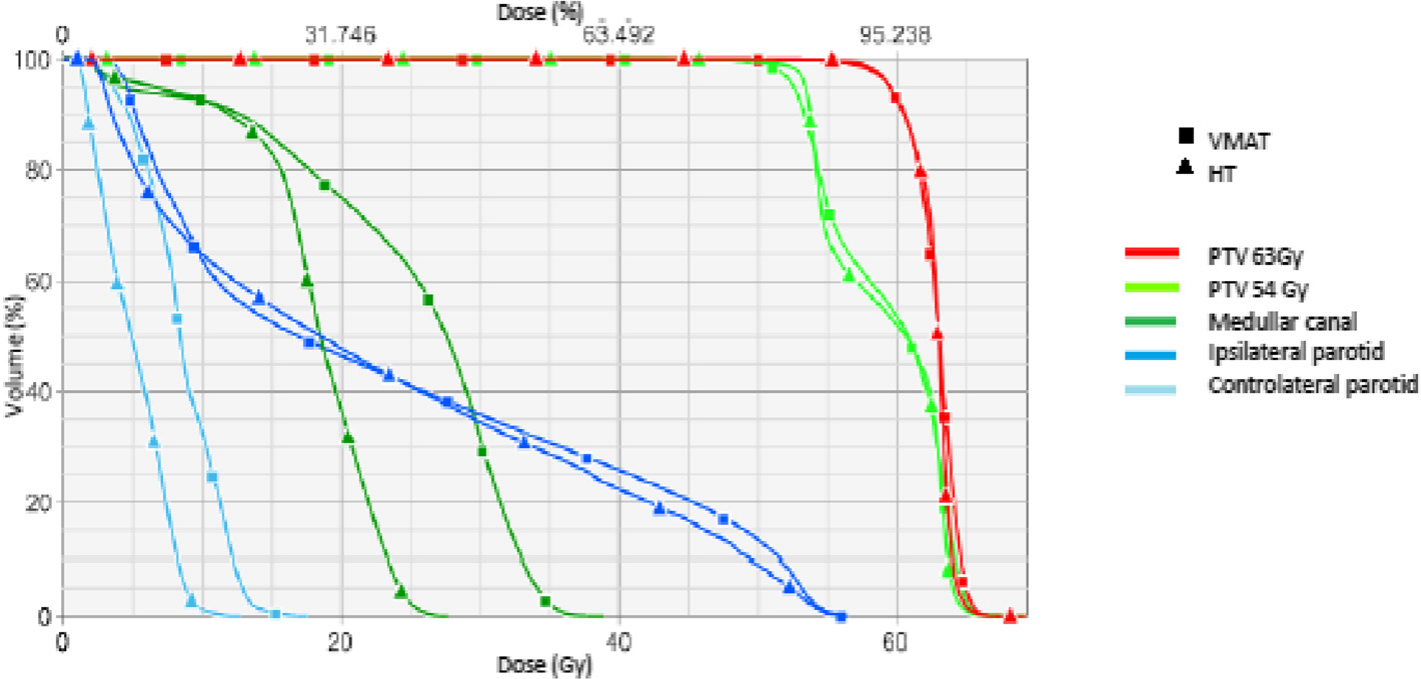
**Dose volume histogram of PTV63 Gy, PTV54 Gy, medullary canal, ipsilateral and controlateral parotid for one representative patient using VMAT and HT.**

These results are illustrated in Figures 1 and 2.

## Discussion

To our knowledge, no previous study compared VMAT and HT specifically for thyroid cancer treatment.

We found that HT for non anaplastic-thyroid cancer provided statistically better tumor coverage index and homogeneity index in comparison with VMAT essentially for low risk PTV. On the other side, we found poorer results with HT for healthy tissue conformity index, conformation number and Dice Similarity Coefficient for low risk and for high risk PTV. Regarding OAR, by comparison with VMAT, HT mainly statistically decreased the maximum dose to the medullary canal.

VMAT and HT represent the ultimate evolution of IMRT since they both allow highly modulated treatments. However, few data are available regarding their direct comparison in the anatomical sites of thyroid cancer irradiation: i.e. median and low cervical regions and upper mediastinum.

For oropharyngeal cancer, several studies are available comparing HT and VMAT. Clemente et al. compared in eight patients also VMAT with both IMRT and HT for a three-dose-level prescription. The same benefit was found for HT compared to VMAT in target coverage and dose homogeneity, but also in conformity [32]. However, the authors did not use the same indexes. Indeed, the conformity index referred to the ratio between the volume of the reference isodose of the low risk PTV and the volume of all PTV (RTOG conformity index). This last index presents a major drawback: it does not take into account the degree of spatial intersection of the two volumes, and is rather adapted to stereotactic radiosurgery. For this reason, we decided not to use this index. Van gestel et al. found that compared to VMAT RapidArc®, HT provided better homogeneity index for high risk PTV but poorer homogeneity index for low risk PTV. They also found that for high risk PTV (but not for low risk PTV), HT provided poorer conformity index than VMAT RapidArc® (defined as our healthy tissue conformity index), that quite agrees with our results [33]. In another study, VMAT RapidArc® was surprisingly globally better in terms of homogeneity index for low risk and intermediate risk PTV and DSC for intermediate and high risk PTV [31].

No study compared VMAT and HT for hypopharyngeal/laryngeal cancers.

Finally, only one study compared VMAT and HT among patients with superior mediastinal tumors (early stage mediastinal Hodgkin’s Lymphoma treated with 30 Gy Involved Nodes Radiation Therapy). The authors found a better homogeneity index for HT and similar results for Conformation Number and for OAR sparing (breast, lung, heart, thyroid gland) in terms of both Dmean and volumes receiving low doses to high doses. (Only a decrease in lungs V10Gy and V15Gy was shown in HT). [34]

As no study compared precisely these techniques for thyroid cancers, this prompted us to perform this dosimetric comparison. We took a particular attention to perform the most credible comparison: all contours were approved by a single physician and all plans were generated by a single physicist, both strongly experimented in both VMAT and HT.

Our results compared to VMAT seem to be coherent with physical characteristics of helical tomotherapy dose distribution. First, opposed to IMRT step-and-shoot characterized by multi-angular beams, HT and VMAT can adopt 360° rotation, which allows delivering a more homogeneous dose in the target area. Nevertheless, in order to improve dose sparing to an OAR or an area, it should be noted that VMAT can use partial gantry rotation with avoidance sectors. In the same way, to achieve this dose sparing goal, HT cannot use partial arcs but it can use the directional block technique, where the beams are forbidden from entering through the defined area but can exit through it. These techniques (avoidance sectors and directional block) are rather adapted to lateral target volumes, and therefore have not been used in this study of thyroid cancers (with concave median target volumes). Besides, HT is equipped with a binary MLC with very fast leaf transition times achieved by using a compressed air system. This combined with the helical fashion in which HT delivers radiation allows a greater degree of intensity modulation compared with VMAT: it could thus explain a more homogeneous dose in the target area and a better coverage of the target as we showed. The HTConf-I refers to the extent to which the reference isodose diverges from the target volume. As far the poorer results for HTConf-I in high risk PTV, this could be explained by differences in optimization techniques: contrary to HT, we defined for VMAT several optimization sub-volumes (“PTV54 – PTV 63” and “PTV54 – (PTV63 + 1 cm)”) to control the dose gradient between high dose and low dose PTV. A way to improve this index in HT could be the creation of a “ring” around the high dose PTV with specific optimization criteria. The poorer results for HTConf-I in low risk PTV could be explained by the process of HT dose delivery. Indeed, the fixed filed size and the lack of dynamic jaws in most helical tomotherapies (unlike new generation of tomotherapy) lead HT to deliver a relatively larger “dose spread” in the superior–inferior direction.

In this respect, normal-tissue integral dose is a burning issue with HT and represents a classical disadvantage of this technique. Indeed, as we reported here, several data suggest that HT increases NTID and healthy tissue volumes irradiated with low dose radiation compared to VMAT [31,35], with an undetermined risk of second cancer. Nevertheless, a few data suggest that new dynamic jaws HTs could now decrease the integral dose compared to regular HTs [36]. Furthermore, even with non-dynamic jaws HTs, it should be noted that a smaller field width (1 cm instead of 2.5 cm as used in our study) could also reduce the normal tissue dose spread, but at the cost of an increased treatment time. (However, due to the relatively small volumes of PTV in thyroid cancer radiotherapy, the treatment time might not be dramatically increased).

Finally, all these data taken together can be confusing to choose between VMAT and HT for thyroid cancer treatment.

Indeed, in all the previous dosimetric studies mentioned, as in ours, the dosimetric differences linked to dose distribution within or just around the PTV are slight, and are probably likely to have no clinical effect in term of local control.

However, the main result of our study is represented by a statistically significant strong decrease in the D2% to medullary canal and its PRV. And this data can be of the utmost importance for several reasons.

Firstly, compared to locally advanced HNSCC patients, patients with advanced thyroid cancers and adjuvant EBRT present prolonged survival regardless of the technique (3D vs. IMRT) [13,16,20,25,26]; therefore, late toxicities have to be considered to choose the best technique, and HT could be interesting in this way. Secondly, patients with non-anaplastic thyroid cancers with indication of EBRT always present aggressive disease with high risk of loco-regional relapse and even of distant relapse (like vertebral metastatic relapse). These patients can thus draw benefits from another irradiation which can be problematic in term of previous dose to OAR, and precisely spinal cord. Finally, among Hodgkin Lymphoma survivors, an estimated 5- to 15-fold increased risk of radio-induced thyroid cancer compared with the normal population has been reported, especially among female treated at a young age [37–41]. These patients may also dramatically beneficiate from a decreased D2% to the spinal cord with HT.

Concerning parotid and sub-maxillary gland sparing, we found a slight decrease in Dmean to the controlateral glands in HT. However, this benefit is difficult to appreciate since salivary glands are not crucial OAR in thyroid cancer irradiation, except when level II has to be treated in the high dose PTV. Besides, we could note that our good results in parotid sparing (in VMAT or in HT) are widely explained by the fact that only two patients had level II nodes in high risk PTV.

We must note however that better OAR sparing with HT is obtained only for high priority penalty OAR, with poorer results for mandible which priority penalty is low, as also reported by Clemente et al. [32].

Last, these results on better high priority OAR sparing are not in contradiction with the poorer healthy tissue conformity index in HT. Indeed, the better gradient dose of HT, the better dose homogeneity and the other characteristics mentioned above lead to strictly respect high priority objectives but at the cost of a large dose spread, with thus poorer healthy tissue conformity.

## Conclusion

In this study, twelve patients with aggressive non-anaplastic thyroid cancer were planned with both VMAT and helical tomotherapy.

Both techniques provided globally attractive treatment plans although slight differences were found in terms of homogeneity or target coverage in favor of HT, or in terms of healthy tissue conformity, conformation number or DSC in favor of VMAT. These slight differences have probably no clinical impact on local control.

However, better medullary canal sparing was obtained with HT, which can be widely interesting in case of re-irradiation, for patients with aggressive disease with high risk of loco-regional relapse or among previously irradiated Hodgkin Lymphoma survivors. Therefore, we think that, if possible, HT should be preferred in case of non anaplastic thyroid cancer radiotherapy.

## Annex 1. TNM staging for non-anaplastic thyroid cancer (7th edition)

T categories:

**TX:** Primary tumor cannot be assessed.

**T0:** No evidence of primary tumor.

**T1:** The tumor is 2 cm across or smaller and has not grown out of the thyroid.

**T1a:** The tumor is 1 cm across or smaller and has not grown outside the thyroid.

**T1b:** The tumor is larger than 1 cm but not larger than 2 cm across and has not grown outside of the thyroid.

**T2:** The tumor is between 2 cm and 4 cm across and has not grown out of the thyroid.

**T3:** The tumor is larger than 4 cm or it has begun to grow a small amount into nearby tissues outside the thyroid.

**T4a:** The tumor is any size and has grown extensively beyond the thyroid gland into nearby tissues of the neck, such as the larynx, trachea, esophagus, or the nerve to the larynx.

**T4b:** A tumor of any size that has grown either back toward the spine or into nearby large blood vessels.

## Abbreviations

CN: Conformation number
CTV: Clinical target volume
DSC: Dice similarity coefficient
EBRT: External beam radiotherapy
HI: Homogeneity index
HT: Helical tomotherapy
HTConf-I: Healthy tissue conformity index
ICRU: International commission of radiation units and measurements
ID: Integral dose
IMRT: Intensity modulated radiotherapy
MLC: Multi-leaf collimator
NTID: Non-tumoral tissue volume integral dose
PRV: Planning organ at risk volume
PTV: Planning target volume
RAI: Radioactive iodine
TCov-I: Target coverage index
VMAT: Volumetric modulated arctherapy
